# Chronic and integrated care in Catalonia

**DOI:** 10.5334/ijic.2205

**Published:** 2015-06-29

**Authors:** Juan Carlos Contel, Albert Ledesma, Carles Blay, Assumpció González Mestre, Carmen Cabezas, Montse Puigdollers, Corine Zara, Paloma Amil, Ester Sarquella, Carles Constante

**Affiliations:** Chronicity Prevention and Care Programme. Ministry of Health; Interministerial Social and Health Care and Interaction Plan (PIAISS). Ministry of the Presidency; Chronicity Prevention and Care Programme, Ministry of Health; Chronicity Prevention and Care Programme, Ministry of Health; Catalan Public Health Agency of Catalonia; CatSalut. Commissioning Health Authority in Catalonia; CatSalut. Commissioning Health Authority in Catalonia; Chronicity Prevention and Care Programme, Ministry of Health; Interministerial Social and Health Care and Interaction Plan (PIAISS), Ministry of the Presidency; Directorate-General for Health Research and Planning, Ministry of Health

**Keywords:** chronic care, expert patient, integrated care, multimorbidity

## Abstract

**Introduction:**

The Chronicity Prevention and Care Programme set up by the Health Plan for Catalonia 2011–2015 has been an outstanding and excellent opportunity to create a new integrated care model in Catalonia. People with chronic conditions require major changes and transformation within the current health and social system. The new and gradual context of ageing, increase in the number of chronic diseases and the current fragmented system requires this transformation to be implemented.

**Method:**

The Chronicity Prevention and Care Programme aims to implement actions which drive the current system towards a new scenario where organisations and professionals must work collaboratively. New tools should facilitate this new context- or work-like integrated health information systems, an integrative financing and commissioning scheme and provide a new approach to virtual care by substituting traditional face-to-face care with transfer and shared responsibilities between patients, citizens and health care professionals.

**Results:**

It has been observed some impact reducing the rate of emergency admissions and readmission related to chronic conditions and better outcome related to better chronic disease control. Some initiative like the Catalan Expert Patient Program has obtained good results and an appropriate service utilization.

**Discussion:**

The implementation of a Chronic Care Program show good results but it is expected that the new integrated health and social care agenda could provoke a real change and transformation. Some of the results related to better health outcomes and a decrease in avoidable hospital admissions related to chronic conditions confirm we are on the right track to make our health and social system more sustainable for the decades to come.

## Introduction–background and problem statement

Catalonia has good health care coverage of proven quality and recognised for its results and the satisfaction of the community that uses these services. However, World Health Organization indications, the current economic downturn and population forecasts that anticipate significant ageing of the population in the coming decades, suggest that the available care models should be reviewed to make the necessary changes, and respond correctly to the challenges of the new social, economic and demographic scenario.

In Catalonia we are facing new challenges with an in-depth population ageing process compared with other European countries. In 2050, over 30% and 12% of the population will be over 65 and 80 years old, respectively. As a consequence, an increasing number of people with chronic conditions will increase very intensively. Currently, 17% and 4.4% of the population are over 65 and 80 years old, respectively [1].

In the past, Catalonia has developed a very good network of primary care centres and long-term care facilities providing very good care in the community as an alternative to hospital care. The split between commissioning and provision role has been incorporated, establishing new contracts and a new commissioning process incorporating cross-cutting targets related to different providers (primary, hospital, mental health and long-term care facilities). However the task to remove organisational silos is a difficult work to perform. More emphasis on integrated care is great opportunity to improve performance.

Within this context, the Ministry of Health of the Government of Catalonia created a Chronicity Prevention and Care Programme at the end of 2011 with an integrated care vision within the new Health Plan, under Government management, explicitly entrusted by legislators to develop this programme and make it operational, in conjunction with the Ministry of Social Welfare and Family. This document is thus the result of a joint effort and the expression of the will to develop it jointly.

Chronicity is a challenge to all developed countries, so the Chronicity Prevention and Care Programme has collected experiences from other geographical areas in our region and adapted them to our local realities. This Programme is set in the framework of the strategic projects and the Health Plan for Catalonia 2011–2015, which makes chronic conditions care a cornerstone of the health system over the coming years and the main driver to achieve integrated care.

It is very important to emphasise the major opportunity from the launching of the new Health Plan for Catalonia 2011–2015 where a new transforming health plan is set out, which aims to attain better results for the Catalan population.

Earlier health plans basically were a large collection of national health targets to be achieved in the next 5-year period. However, the Health Plan for the period 2011–2015 describes expected changes and transformations which adapt the model to the forthcoming challenges in future years. It has been very important to integrate our new Chronicity Prevention and Care Programme into the new Health Plan, being a real Strategic Plan, for the next few years.

The literature review contributes very good examples of transformation such as Kaiser Permanente, the Veterans Health Administration, and high performing regional experiences like the Strategy to Tackle the Challenge of Chronicity in the Basque Country have been very inspiring models to extract and adapt some key features [2–7].

Some interesting key drivers have been identified as high inspiring points to be incorporated in our model: chronic and integrated care policy-driven orientation, introduction of stratification, commitment of clinical leadership involved in design and implementation of local integrated care pathways (ICPs), shared ICT between clinicians and between patients and professionals, overcome of financial barriers introducing new joint cross-cutting targets among primary and secondary care, community care orientation promoting more care at home avoiding unnecessary emergency admissions and institutionalisations and self-management policies.

In addition, the Spanish Ministry of Health has drawn up a national chronic care strategy published 1 year ago with some common principles and aims although the Catalan Ministry of Health has decentralised and total competences to prepare and implement its own chronic care programme [8].

## Need and description of various policy developments

**Figure 1.**
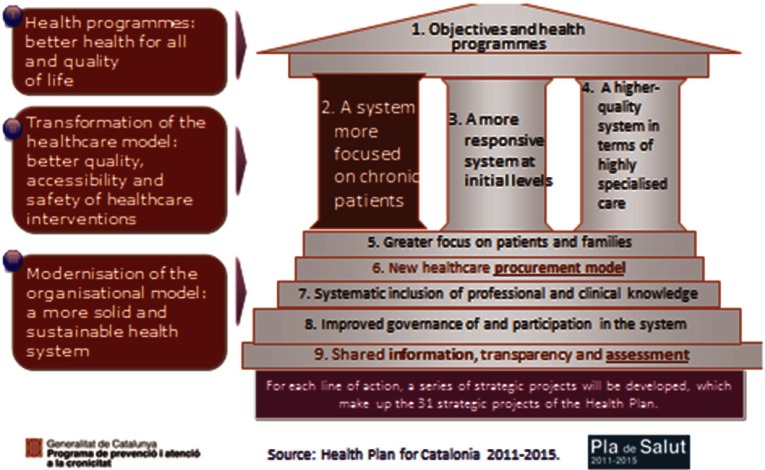
Health Plan for Catalonia 2011–2015

The Health Plan for Catalonia is one of the most important instruments prepared by the Government of Catalonia. It has 32 strategic projects, 6 of which are related to the Chronicity Prevention and Care Programme. This new Health Plan introduces the following areas of work (Figure 1).

An ‘integrated care’ vision within the health sector but also including an initial collaborative work with social services. Chronic care is expected to be a driver to promote an integrated health delivery approach.

New contractual and financial scheme to incentivise integrated care incorporating some cross-cutting targets related to all providers performance. All agencies are called to contribute to joint targets.

A more interactive and inter-operative global health information system (HIS) through the Shared electronic Health Record of Catalonia (eHR) and the Personal Health Channel (‘Canal Personal Salut’), which facilitates remote care and direct access by patients and citizens. Commissioning authorities are urging all providers to publish a minimum data set of information and reports like hospital discharge report, structured diagnosis generated, structured clinical variables.

Population stratification using clinical risk groups (CRGs) by 3M enterprise to support clinicians to identify people who could be at risk of hospitalisation, readmission or death. Analytical Services at the Catalan Ministry of Health is providing stratification for all providers and it is published in the eHR to be shared by all clinicians. Now this Analytical Services is developing home own made stratification model.

Some information system tools have been introduced to monitor indicators related to this Programme, especially avoidable emergency admissions related to ambulatory care sensitive conditions (ACSC), based on American Healthcare Research and Quality Agency (AHRQ), 30-day readmissions related to the main chronic conditions, updated monthly and accessible to providers and observed chronic conditions prevalence identified by providers.

In addition, the Chronicity Prevention and Care Programme sets out different actions for an increasing number of populations with concurrent health and social needs, especially complex chronic patients (CCP) with multimorbidity or advanced chronic disease (ACD) with social needs or dependence.

**Figure 2.**
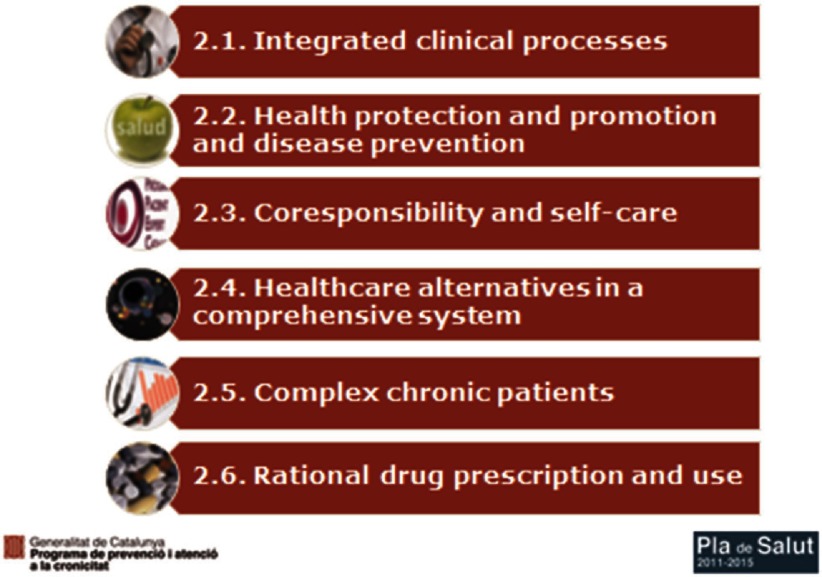
Strategic lines of the plan

The Chronicity Prevention and Care Programme operates within the following basic work areas (Figure 2; Table 1) [9]:

Developing comprehensive clinical processes redesign for the chronic conditions with the greatest impact in all areas by building ICPs in each geographical area which comprises a hospital, primary care centres, nursing home facilities and a mental health network. Clinical leaders are called by commissioning authorities to be incorporated in this process of generating local agreements regarding singular provision in each area. It is a joint work performed by health authorities and clinicians belonging to different sectors. Therefore, ICPs based on local clinical facilitate real implementation.

Strengthening health protection, promotion and prevention as instruments for maintaining health and preventing chronic disease [10]. In addition, a new strategy called PINSAP has been launched where key elements of public health are being introduced in cross-cutting governmental policies established by other ministries such as the ministries of Education, Social Welfare and Family, and others.

Promoting the self-care and personal responsibility of citizens concerning their health, risk factors or diseases. The successful Expert Patient Programme Catalonia (EPP) has been implemented with over 4000 patients included in the Programme to date. This Programme comprises an structured methodology where “experts and trained patients” coach and lead equals and comprises different chronic diseases such as diabetes, COPD, heart failure, dementia carers.

Deploying social services and health care facilities working in a more integrated care approach, and adequate comprehensive systems for providing care for chronic and dependent patients. As a consequence, it has been launched a new integrated health and social care plan in Catalonia since March 2014 where it is expected real integrated care between health and social services. The chronic care approach requires an updated vision incorporating social care contribution to achieve better care for people with complex health and social care needs. This new Plan has created great expectation to overcome some of the barriers we have identified in the Chronicity Prevention and Care Programme.

Providing comprehensive and proactive care of patients with complex chronic disease and ACD that ensures a 24/7 coverage model with a good response to potential exacerbations of this patient group. Most ICPs are well designed in a day care approach but they fail during nights and weekend time. Therefore we have reinforce and commissioned each territory agree and incorporated in the pathway, written statements about how to guarantee a quick response in case of acute exacerbation. Different practical actions have been covered including this 24/7 guarantee.

Rationalising the use of medications, especially with people with polypharmacy and improvement of adherence in chronic patients. This is an area where pharmacists have been involved in the construction of a better ‘complex care model’.

Promoting an alternative remote care model substituting face-to-face visits with virtual contacts like telephone and electronic messaging. Citizens have been invited to access to a National Personal Health Folder through ‘robust password’ instead of ‘digital certificate’. Since September 2014 the Catalan Ministry of Health is promoting this strategy to involve an increasing number of citizens and patients.

Replacing acute conventional hospitalisations with other alternatives: sub-acute facilities, day care facilities, a more proactive home care programme in primary health care. It is expected a number of acute hospital emergency admissions could be substituted by these friendlier and more cost-effective alternatives.

In this context, health and social services face the challenge of transforming the current health care model, adapting it to meet the needs of citizens and doing so in a way that is sustainable for the system. It should be kept in mind that many clinical conditions that lead to situations of chronicity and dependence can be prevented or delayed, thus delaying disability for later stages of life or, in other words, increasing a person's disability-free life.

The new Interministerial Social and Health Care and Interaction Plan (PIAISS) introduce real integrated care approach to overcome this challenging issue and develop a real ‘person-centred care model’.

Transforming the model involves not only improving the skills of the professionals and all workforce involved in care processes, but also redesigning how services are provided and encouraging cooperation between health care and social service sectors, and between organisations and professionals working for the same patients.

In addition, some new financial and contract scheme has been introduced in 2014 to modify and transform the current health provision. Common and cross-cutting targets and indicators such as the following are being introduced. It is expected that professionals working at different areas (primary care, hospital, mental health and long-term care facilities) have a shared outcome framework as an important driver which facilitates and reorient their performance [11]. Here there is a description of these targets which have been incorporated in all contracts:

- To decrease taxes on avoidable emergency admission of ACSC using both a ‘composite’ and specific diseases indicators (COPD, heart failure).
- To decrease the 30-day readmission rate using both a ‘composite’ and specific diseases indicators (COPD, heart failure).
- To achieve a minimum rate of prevalence of CCP and ACD who require a palliative approach. A basic and published intervention plan (IP) generated in a “key information summary” has been elaborated and uploaded to the eHR, which is accessible to all providers, and especially emergency services, ranging from the 061 emergency call centre to emergency services. When a patient call during an exacerbation in a 24/7 scheme, a ‘warning alert’ activates staff in the room and key information summary is accessible to professionals working in the call centre to facilitate basic information to take the best decisions. Additional information could be seen in the national eHR.

It is expected to introduce a new triple-aim approach to the contract as a ‘powerful integrator driver’ in the next years. There are no measures related to ‘experience of care’ domain [12].

### Initial results related to some strategic lines

Two years after the implementation, it has been achieved some initial results which encourage reinforcing this strategy and new key actions are being incorporated to strengthen this journey towards more integrated care progress.

It could be reported some progress in the following areas:

#### Integrated care pathways related to four chronic conditions

A conceptual and methodological framework with a related implementation-oriented checklist has been used on a local level to design and later implement these pathways. This action is perhaps the most important strategy to achieve ‘bottom-up’ change and transformation. Managers and clinical leaders wanted to have available practical guidelines to support implementation process.

Over 80% of ‘natural territories’ or ‘microsystems’ have drawn up and implemented, at least in a preliminary way, the ICPs related to four clinical conditions: diabetes mellitus, heart failure, COPD and depression. All territories have ICPs for three clinical conditions. We consider a ‘microsystem’ as a territory or geographical area where most chronic patients use services required by them. In general terms, this includes a hospital with secondary care services, primary care teams operating collaboratively with the hospital, a nursing home and mental health and social care.

New ICPs will be designed and implemented related to additional clinical conditions: dementia, chronic kidney disease, chronic osteoarticular pathology and chronic pain.

#### Promoting self-care on a community level

**Figure 3.**
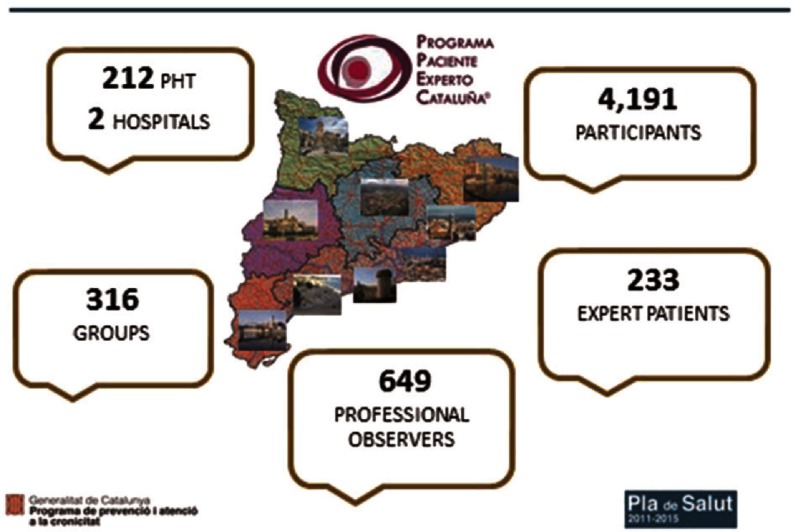
Catalan Expert Patient Programme

One of the most successful projects is the ‘Expert Patient Programme Catalonia®’ (EPP). The most important features are the following: (Figure 3) [13]

- Over 4000 patients and 316 groups have participated in the Programme; 233 people have transformed into ‘expert patient’ leading groups.
- A total of 212 primary care teams are involved in the Programme. Almost 60% of the teams and three hospitals are taking part in the project. Different public and non-profit providers are also involved.
- A total of 649 professionals, 376 nurses, 30 social workers and 178 family doctors have participated as observers.
- Good results have been achieved concerning improvement of all measures evaluated: knowledge, habits and lifestyles, self-care and quality of life, use or services including emergency hospital admission and use of A&E visits. These improvements are still maintained 6–12 months after groups have finished.

In the case of heart failure and COPD, there is a reduction of 37% and 42% of emergency hospital admissions, respectively.

Next steps in the Programme will be the elaboration of two new forthcoming guides in depression and obesity, and a new project called ‘Expert Carer Programme Catalonia’ related to four areas of caring support: children with chronic diseases, patients with dementia, CCP and patients with a severe mental disorder.

Another work area is the improvement of ‘health literacy’. There has been incorporated into the Health Survey for Catalonia (HSC) administered twice a year to 3000 Catalan citizens, questions related to health literacy. During 2014 the HCS included a specific module version in Catalan and Spanish, the short version of the European Health Literacy Survey Questionnaire (HLS-EU-Q16). This 16-item questionnaire is an abbreviated version of questionnaire HLS-EU-Q47 drawn up by the HLS European Health Literacy project 2009–2012 [14]. We could assess the current ‘health literacy’ situation in the future and progression over the next few years.

#### Care of patients with complex chronic conditions

**Figure 4.**
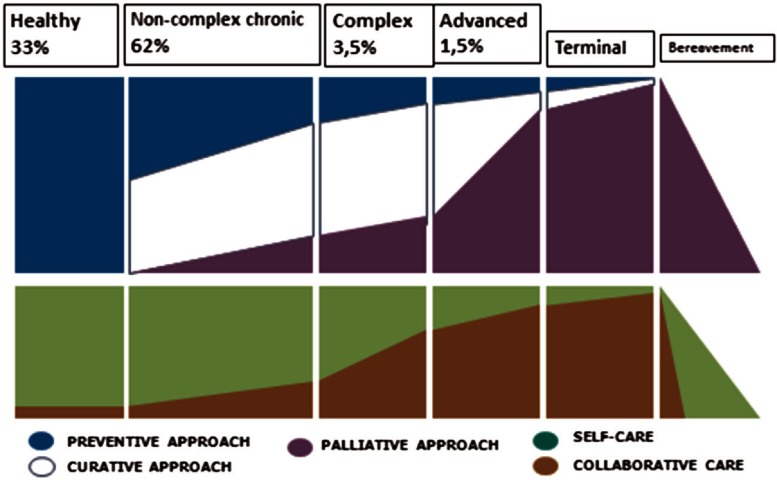
The continuum of chronicity

This implies a very challenging area of work because of the greater needs of patients with these conditions; a model has been created on a local level more oriented to coping with the enhanced needs and demands generated by frequent acute exacerbations and intensive use of services (Figure 4) [2, 15, 16].

**Figure 5.**
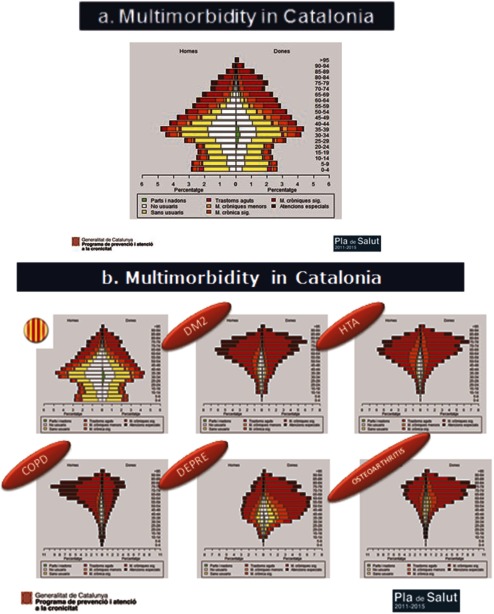
Multimorbidity in Catalonia

**Figure 6.**
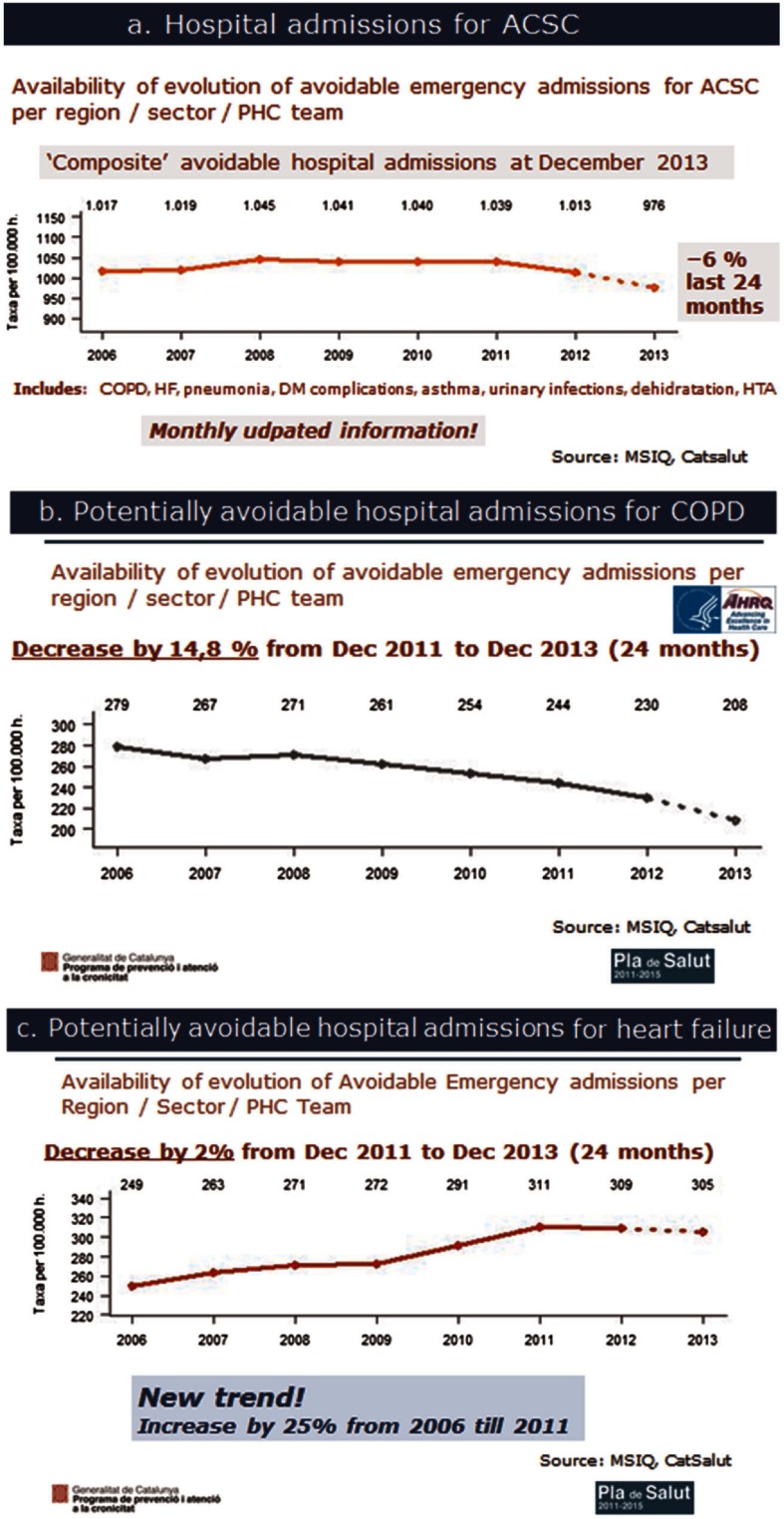
Potentially avoidable hospital admissions for (a) ACSC, (b) COPD and (c) heart failure

However, this change and transformation requires time; the following initial results and milestones related to implementation of this project can be identified:

- The possibility of uploading to the eHR a new launched and ad-hoc ‘complex chronic condition’ and ‘advanced chronic disease’ mark or label to be available to all providers and professionals who work and act in care of these patients has been set out. This mark has an attached individual minimum IP as a key information summary with critical and summarised information of the patient-like diagnosis, pharmacy, services used and ‘what to do recommendations’ in case of a ‘crisis or exacerbation’ and ‘advanced care planning’ directives. After 18 months of this development, we have 109,000 patients marked with this condition, approximately 1.5% of the Catalan population.
- A stratification model has been developed in Catalonia by means of CRGs supported by 3M. The entire Catalan population is classified in a different morbidity group and some ‘score risk’ to be admitted to hospital in the next 12 months is set out. This work has been able to have been performed by the availability of all minimum data sets aggregated related to primary health care, hospital, nursing homes and mental health and pharmacy. Elaboration of the stratification includes giving different functionalities: (a) adjustment of the simulation budgetary assignment model for the forthcoming 2015 new financing scheme, (b) information published at eHR about individual morbidity group classification and ‘risk scores’ related to future potential risk of hospitalisation and death, which are all very important to identify CCP and advanced chronic patients respectively (c) to return stratified population databases to the different providers so they can incorporate this information and manage it in the different local HIS and deliver to front-line clinicians. Interesting developments are introduced in some HIS with the possibility of each clinician editing a list of patients at high risk and commencing proactive care management (Figure 5a and b) [17, 18].
- The number of emergency admissions related to chronic conditions (COPD, heart failure, asthma, diabetes complications, etc.) has decreased from 1039 per 100,000 inhabitants at January 2012 to 976 in December 2013 – a decrease of 8% in 24 months (Figure 6a, b, and c) [11].
- We have reduced the number of emergency admissions related to COPD from 244 admissions per 100,000 inhabitants to 208 in December 2013 – a decrease of 14% in 24 months.
- We have reduced the number of emergency admissions related to heart failure from 311 admissions per 100,000 inhabitants to 305 in December 2013 – a decrease of 2% in 24 months. Up until 2011 it has been an increasing tendency with an increase of 25% from 2006 until 2011 with a turning point since then.
- The number of people with diabetes complications has been reduced from 63 admissions per 100,000 inhabitants to 59 in December 2013 – a decrease of 6%.
- The 30-day readmission rate stayed stable around 11% with a discrete decrease during the same 2-year period.
- Introduction of an increasing number subacute services beds with less intense care such that it enables offering a very good alternative to acute conventional hospital care for CCP. These facilities are directly accessible from primary care teams.
- Gradual improvement of home care and day care facilities in the community as a good alternative to conventional hospital care, accessible to primary care professionals.
- A mental health programme is being incorporated into pathways to agree on an approach to CCP with mental disorders.

#### More rational use of medicines

Guidelines that determine the implementation of a cross-cutting programme of rational use of medicines and incorporate support tools for a more appropriate prescription have been designed.

There is an emphasis on improvement in the regular and systematic review/conciliation and adherence with the chronic medication and, in particular, in complex patients.

Some important achievements have been made:

- A new methodological document denominated rational drug use. Medication management in the complex chronic patient: reconciliation, revision, deprescription and adherence have been released. An online course has been prepared for clinicians.
- A new instruction has been launched by the commissioning authority CatSalut that sets out minimum criteria for good prescription, review and conciliation.
- Drawing up of conciliation protocols among local providers.
- A new quality indicator to assess ‘secure prescription’ in CCP has been created. This indicator is evaluated by CatSalut by periodically counting and monitoring the taxation of security incidences per patient.
- A new ‘prevention and care of chronic patients from community pharmacies’ has been drawn up. An increasing number of ‘individual and personalised dosage’ initiatives are being implemented to improve security.
- New ‘harmonised therapeutic guidelines’ have been launched to improve prescription criteria related to chronic conditions.
- The patient can access their own medication plan by means of the Personal Health Channel platform to refill prescriptions.
- Until now, over 85% hospitals and 30% mental health centres have incorporated the electronic chronic prescription. This enables better possibilities for clinical and electronic conciliation in the community. Virtually 100% of primary care providers work with electronic prescriptions.
- Some new deprescription initiatives have been commenced with advanced chronic patients because of the rationalisation required and appropriate prescriptions based on a palliative approach to manage these patients.

#### Health promotion, protection and prevention of chronic diseases

One of the most important features of the Chronicity Prevention and Care Programme in Catalonia has been to incorporate a strong emphasis on health promotion and disease prevention. This could be described as the most important development in this area [19]:

- A total of 140,000 people have stopped smoking during 2-year period. A comprehensive and cross-cutting approach from prevention of the onset of smoking to smoking cessation programmes aimed at people with chronic diseases (e.g. respiratory and mental diseases) has been put in place. In addition, a new pioneering regulation in Catalonia to prohibit the use of electronic nicotine delivery systems has been launched. More than 1000 health care professionals have been trained with innovative online courses.
- More than 120,000 people have improved their *physical activity* level, moving from a sedentary to an active lifestyle during 2-year period. All primary care centres have been included in a strategy that uses physical activity advice from health professionals and identifies the community resources available (‘healthy walks’).
- A ‘Mediterranean-labelled diet’ (AMED) has been introduced in 290 establishments that served more than 44,000 guests every day.
- Strategies have been implemented to decrease alcohol intake; a continuing education programme has been developed for primary care professionals within the so-called drink less (‘Beveu Menys’) programme to promote stopping high alcohol intake.

## Discussion

In general terms, the Chronicity Prevention and Care Programme set up by the Health Plan for Catalonia 2011–2015 have contributed to create better conditions to achieve better outcomes for chronic patients. It has been observed some better figures related to a range of outcomes established at the end of 2011. In addition to these results, it should be recognised an important progress to redesign the model of care, especially in Catalonia where a mixed provision coexists.

We would like to emphasise some positive achievements to be remarked as *facilitators*:

- Strong governmental leadership and commitment through the Health Plan and the Chronicity Prevention and Care Programme. They have been a catalyst to encourage real integrated health care.
- It has been recognised the need to reinforce a strategy of joint managerial and clinician leadership. Hidden clinician talent has emerged and incorporated in the strategy.
- Complex chronic care as a strong focus to align commissioning authorities, providers and clinical leaders.
- Combination of both care model redesign and instrumental tools such as integrated information system and commissioning and contract policy. One strategy reinforces the other.
- ICPs have been an opportunity to achieve local agreements between clinicians as a guarantee of real implementation.
- Incorporation of the 24/7 vision, involving emergency services in overall strategy to take best decisions in an out-of-hours scheme for acute exacerbations in complex patients.
- It has been experienced some integrated health and social care pilots identifying areas of improvement for the PIAISS.
- The Chronicity Prevention and Care Programme team has worked closely with both the Information System Unit and the Commissioning Unit at the Ministry of Health to coordinate implementation strategy.

Otherwise, we have identified some barriers. They have been analysed and some actions are being incorporated in the PIAISS. We could describe the following barriers:

- These programmes need time and patience. Recognised success stories take a lot of time to be implemented and obtain good results.
- Hospital activity-based payment should be changed. A reform of financing scheme will be launched in year 2015 transferring in some demonstration projects some budget from hospital to primary care to reinforce primary care high performance resolution.
- Social services should be more involved in the integrated care strategy. The PIAISS will work in the care model redesign aligning information system and commissioning strategy at both the Ministry of Health and the Ministry of Social Welfare and Family.
- Commissioning bodies should incorporate intensively more clinical involvement such as Clinical Commissioning Groups in England. A balanced managerial and clinical approach is expected in order to guarantee real implementation.
- Commissioning authorities must oblige all providers to upload a key information summary in the national eHR. New contractual targets are being incorporated regarding information system terms.
- There should be more alignment between outcome framework and local professional's payment scheme developed by organisations.
- A better comprehension of complex patients should be developed. Current stratification does not explain complexity. New social variables should be included in the stratification process.
- It is not sustainable to maintain two separated health and social commissioning authorities. It is expected the new Plan encourages a new joint health and social commissioning authority.
- There are real problems related to strict regulations in data protection which do not allow to share health and social information in a unique or Shared Health and Social Care Record. New and more national and European regulation must facilitate this important work.

## Conclusions

**Figure 7.**
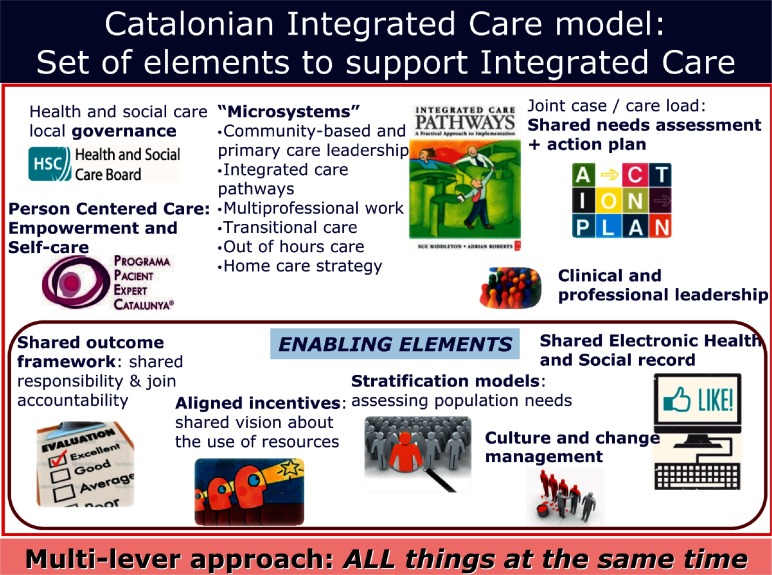
Catalan integrated care model: set of elements to support integrated care

Implementing an integrated care strategy is a long journey and this will take time and patience. A shared vision is required and hard work must be carried out to align all the elements which help and encourage an integrated care scenario (Figure 7).

Our experience generates interesting lessons for other countries and regions who are working in a similar transformational project.

We would like to mention and share some of these lessons:

- The real narrative of integrated care should be based on the construction of a person-centred care model where citizens could be involved in the decision process. This model require to strengthen both primary health and social care as a basic key element of the model.
- Care for complex persons with health and social needs has been an important focus to encourage a more integrated care orientation between all providers and professionals who are working and sharing care for the same people. The best way to involve local providers at local level is to design and implement local ICPs. It is a very good scenario and challenge to gather together clinical, professional and managerial leadership from different areas (primary health care, hospital, mental health, long-term care and emergency services in addition to professionals working in social services now under the PIAISS) to agree on how to take care of people with chronic conditions, and acknowledge that collaborative work is possible and necessary. These pathways are being elaborated on the basis of 24/7 coverage and articulate a good response for crisis and acute exacerbation situations.
- Current eHR should be transformed into a new shared clinical and social care record that incorporates progressively social care information. The new Shared Health and Social Care Record should be constructed to facilitate virtual work and better communication between primary, secondary and social care professionals and new ICT platform tools should give support not only to clinicians but also to patients and citizens with new tools and functionalities to facilitate more virtual work instead of a face-to-face approach. In addition, it should be extended the use of the Personal Health and Social Folder or Channel which facilitates new opportunities to patients and citizens to establish a new relationship between them and professionals with an increasing number of services, promoting virtual communication between patients and citizens with health care and social care professionals. Available aggregate data which facilitate current stratification should incorporate progressively social care data to introduce some predictive modelling for events of special interest in the social care sector like risk of being an higher user of social services or institutionalisation.

In addition, some lessons could be taken related to new trends and innovations in Europe:

- New integrated health and social care models in Europe like in Scotland and England will contribute with interesting and innovative financing schemes which encourage best behaviour both in the health and social care sector to promote real Integrated Care.
- These new models will be based on innovative population-based triple aim vision included in a Shared Outcome Framework, identifying new measures related to ‘experience of care’ dimension.
- Strong commitment of the government and the creation of Health and Social Care Boards is required to plan and commission better Integrated Care at county and municipality level, creating joint commissioning teams.
- Some initiatives of joint Assessment and Intervention Plan tools should be considered to incorporate new methodology to work in a more collaboratively way.
- Finally, no effort should be made towards structural and formal integration. More and real emphasis must be placed on functional integrated care that encourages clinical and service integration at the point of delivery. Energy and efforts should be channelled to improve actual care conditions on a local level for patients and citizens [20–23].
- In Catalonia, work will be based on a functional approach and strategy to achieve integrated health and social care. This strategy will comprise a care model redesign, ICT solutions for health and social sectors, joint commissioning with a population-based triple aim vision and more involvement of citizens to construct a narrative of a person-centred model.

## Figures and Tables

**Table 1.**
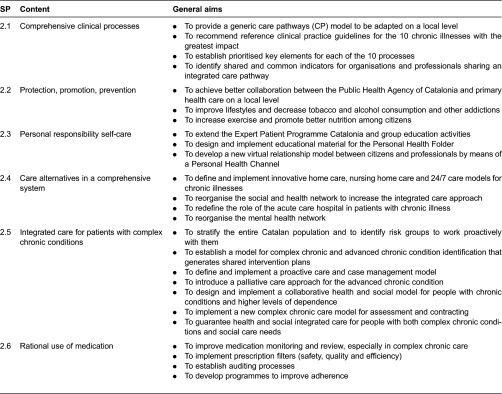
Chronic care programme actions in the Catalan chronic care programme
